# The Role of Uncoupling Proteins in Diabetes Mellitus

**DOI:** 10.1155/2013/585897

**Published:** 2013-06-05

**Authors:** Jing Liu, Ji Li, Wen-Jian Li, Chun-Ming Wang

**Affiliations:** ^1^Institute of Modern Physics, Chinese Academy of Sciences, Lanzhou 730000, China; ^2^Department of Pharmacology and Toxicology, School of Medicine and Biomedical Sciences, University at Buffalo, State University of New York, Buffalo, NY 14214, USA; ^3^School of Life Sciences, Lanzhou University, Lanzhou 730000, China

## Abstract

Uncoupling proteins (UCPs) are anion carriers expressed in the mitochondrial inner membrane that uncouple oxygen consumption by the respiratory chain from ATP synthesis. The physiological functions of UCPs have long been debated since the new UCPs (UCP2 to 5) were discovered, and the role of UCPs in the pathogeneses of diabetes mellitus is one of the hottest topics. UCPs are thought to be activated by superoxide and then decrease mitochondrial free radicals generation; this may provide a protective effect on diabetes mellitus that is under the oxidative stress conditions. UCP1 is considered to be a candidate gene for diabetes because of its role in thermogenesis and energy expenditure. UCP2 is expressed in several tissues and acts in the negative regulation of insulin secretion by *β*-cells and in fatty acid metabolism. UCP3 plays a role in fatty acid metabolism and energy homeostasis and modulates insulin sensitivity. Several gene polymorphisms of UCP1, UCP2, and UCP3 were reported to be associated with diabetes. The progress in the role of UCP1, UCP2, and UCP3 on diabetes mellitus is summarized in this review.

## 1. Introduction

**Figure 1:**
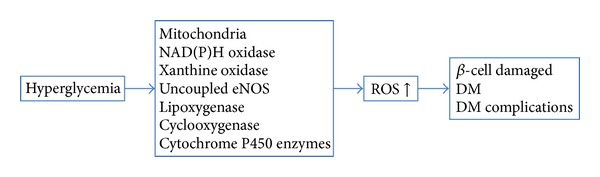
The mechanisms of ROS and DM development. Hyperglycemia may activate ROS generation through the mitochondrial and nonmitochondrial origins: NADPH oxidase, xanthine oxidase, uncoupled eNOS, lipoxygenase, cyclooxygenase, cytochrome P450 enzymes, and other hemoproteins. ROS can further cause *β*-cell damages and the development of DM and related complications.

Diabetes mellitus (DM) is a chronic metabolic disease which is characterized by hyperglycemia, absolute or relative deficiencies in insulin secretion, and/or insulin action. The new classification proposed by the American Diabetes Association in 1997 was based on the pathogenesis of the disease and comprises four categories: Type 1 DM (DM1), Type 2 DM (DM2), other types, and gestational diabetes [1]. DM1 is mostly due to genetic disorders and an autoimmune disease resulting in the selective destruction of *β*-cells in the pancreas that leads to insulin loss. DM2, also called noninsulin-dependent diabetes mellitus (NIDDM), is characterized by insulin resistance with significant metabolic dysfunction including obesity, impaired insulin function and secretion, and increased endogenous glucose output. Although the two types of diabetes have distinct etiologies, they lead to similar diabetic complications, and both of them are related to oxidative stress status. It is demonstrated that activation of oxidative stress pathways plays a key role in the development of not only the late complications (such as cardiovascular disease, nephropathy, retinopathy, and amputations) in DM1 and DM2, but also in the early stage such as insulin resistance [2]. There are many sources of reactive oxygen species (ROS) production in diabetes including mitochondrial and nonmitochondrial origins: NADPH oxidase, xanthine oxidase, uncoupled eNOS, lipoxygenase, cyclooxygenase, cytochrome P450 enzymes, and other hemoproteins [3], and mitochondrion is thought to be the main source of ROS generation site in DM. The mechanisms about ROS and DM development were shown in Figure 1.

**Figure 2:**
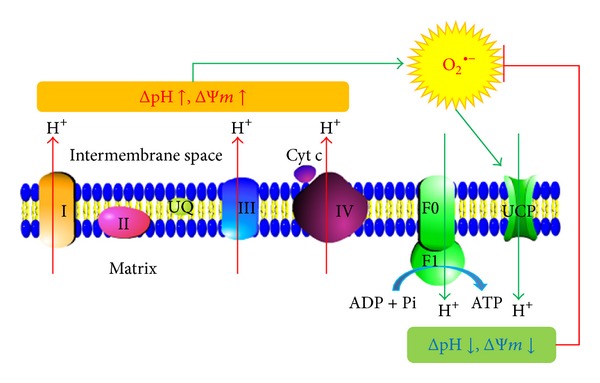
“Antioxidative activity” of UCP. High transmembrane proton gradient and membrane potential of mitochondria will induce ROS production and thus oxidative damage; these ROSs may activate UCPs and therefore cause a “mild uncoupling” and (as a negative feedback) will prevent further superoxide production and decrease oxidative damage. ΔpH: transmembrane proton gradient; ΔΨ*m*: mitochondrial membrane potential.

The uncoupling proteins (UCPs) are a family of mitochondrial transport proteins located in the inner mitochondrial membrane. There are five UCPs (named UCP1 to 5) found in mammals [4]. These anion-carrier proteins transport protons (H^+^) to the mitochondrial matrix and in turn dissipate the proton motive force as heat and uncouple the substrate oxidation from the production of ATP. These proteins have similarities in their structures, but different tissue distributions in mammals. UCP1 is mainly expressed in brown adipose tissue (BAT), which is responsible for thermogenesis in newborns. UCP2 is widely distributed in several tissues including the spleen, kidney, immune system, pancreas, and central nervous system, whereas UCP3 is mainly restricted to the skeletal muscle, and UCP4 and UCP5/BMCP1 are mainly expressed in the brain. Besides the nonshivering thermogenesis function of UCP1, functions of the other UCPs are still unclear. UCP2 is reported to be involved in glucose and lipid metabolism [5] to control immune cell activation by modulating MAPK pathways and the production of mitochondrial ROS [6], and a neuroprotective role is also suggested based on the regulation of mitochondria membrane potential, production of ROS, preservation of calcium homeostasis, modulation of neuronal activity, and eventually inhibition of cellular damage [7]. UCP3 is suggested to be involved in mediating energy expenditure via uncoupling, especially in fatty acid metabolism, and it seems to protect mitochondria against lipid-induced oxidative stress, which makes this protein a potential player in the development of DM2 [8]. Fewer studies focused on the physiologic roles of UCP4 and UCP5, and the protection against oxidative stress and mitochondrial dysfunction are also reported [9]. Although the physiological functions of UCPs are still not been completely elucidated, their abilities of reducing mitochondrial ROS formation are widely accepted [10]. That is, high membrane potential of mitochondria will induce ROS production and thus oxidative damage; these ROS may activate UCPs and therefore cause a “mild uncoupling” and (as a negative feedback) will prevent further superoxide production and decrease oxidative damage (Figure 2). This “antioxidative activity” of UCPs makes it logical to search any benefit on DM through counteracting the oxidative stress appeared in DM and the complications. On the other hand, it is also found that UCP2 is a negative regulator of insulin secretion; the superoxide-mediated activation of UCP2 causes pancreatic *β*-cell dysfunction [11].

The vast majority of diabetes are Type 1 or Type 2 (DM2 represents at least 80 percent and DM1 accounts for about 5–10 percent), and increasing numbers of studies focused on the roles of UCPs on DM or the complications. We will review the progress in the relations between UCPs (UCP1, UCP2, and UCP3) and DM1 and/or DM2 in the following.

## 2. Role of UCP1 in DM

The relationship between UCP1 and DM has already been reported long before the other UCPs were discovered in 1997. Studies revealed that UCP1 mRNA and protein concentrations in BAT were regulated by insulin [12, 13]. As UCP1 has been proved to decrease membrane potential, downregulate ROS generation, and increase energy expenditure, so *UCP1* gene is regarded as a candidate gene for obesity, DM2, or related traits. The role of UCP1 in the development of obesity and DM2 has been reviewed in 2010 and 2012 [14, 15]; the authors focused on the polymorphisms −3826A/G, −1766A/G, and −112A/C in the promoter region, Ala64Thr in exon 2, and Met299Leu in exon 5 of *UCP1* gene and pointed out that they are possibly associated with obesity, lipid/lipoprotein-related disease, and/or DM2. The −3826A/G polymorphism of UCP1 was further reported to be associated with diabetic retinopathy (DR) in DM1 group and the *UCP1* gene expression was increased in human retina [16]; while the same polymorphism of UCP1 (−3826A/G) was not found the association with DM2 with European ancestry in another study [17], although a significant association between the UCP2 Ala55Val and UCP3 −55C/T polymorphisms and increased susceptibility for DM2 were detected in Asians in the same study [17]. The −3826A/G polymorphism influenced *UCP1* gene expression: G allele carriers had higher *UCP1* cDNA and protein concentrations than A/A carriers. And more interestingly, G allele carriers exhibited increased *MnSOD2* expression, which suggested that this allele could be a marker of oxidative stress. As oxidative stress is related to DR, so this deleterious polymorphism in *UCP1* gene is suggested to be a risk factor for DR (multivariate analysis confirmed that the G/G genotype was an independent risk factor for DR) [16].

UCP1 had been thought to be expressed only in rodents and human infants for a long time; however, UCP1 protein and/or its mRNA expression were detected in human white adipose tissue, skeletal muscle, longitudinal smooth muscle layers, retinal cells, and islet cells recently [18, 19], although the physiological functions of UCP1 in these tissues and organs are not established as well as in BAT. In 2013, the adult human neck brown fat is further reported with the anatomical localization, gene expression profiling, and functional characterization [20]. The imbalance between energy intake and expenditure is the underlying cause of obesity and DM. BAT consumes fuel for thermogenesis through tissue-specific UCP1; it was once thought that BAT had a functional role in rodents and human infants only, but it has been recently shown that in response to mild cold exposure adult human BAT consumes more glucose per gram than any other tissues [21]. In addition to this nonshivering thermogenesis, human BAT may also combat weight gain by becoming more active in the setting of increased whole-body energy intake. This suggests that activation of human BAT could be used as a safe treatment for obesity and metabolic dysregulation and further help to cure DM. In view of the *UCP1* gene expressed in the other tissues, more attention may be paid to the role of UCP1 in muscle tissue, islet cells, and thymus function in the future.

## 3. Role of UCP2 in DM

UCP2 is the most widely distributed UCP and highly expressed in pancreatic *β*-cells in DM, so it is the most frequently studied one concerning its role in DM. Mitochondrial dysfunction and *β*-cell failure exhibited a close correlation in DM2. In *β*-cells, ROSs activate UCP2, which results in proton leak across the mitochondrial inner membrane, and this leads to reduced *β*-cell ATP synthesis and content, which is a critical parameter in regulating glucose-stimulated insulin secretion (GSIS) [22]. The recent reviews about UCP2 and DM were published in 2009 and 2011 [23–25]. We will review the progress acquired since then in the following.

### 3.1. UCP2 Is a Negative Regulator of Insulin Secretion

Early in 2001, just 4 years after the discovery of UCP2, it was reported that UCP2 negatively regulated insulin secretion and was a major link between obesity, *β*-cell dysfunction, and DM2 [26]. In a model of obesity-induced diabetes mice (ob/ob mice), UCP2 was markedly upregulated in islets. The UCP2-deficient mice had higher islet ATP levels and increased GSIS; this indicated that UCP2 negatively regulates insulin secretion. The ob/ob mice lacking UCP2 had restored first-phase insulin secretion, increased serum insulin levels, and greatly decreased levels of glycemia [26]. In recent years, the mitochondrial ROS generation or oxidative stress in *β*-cell was found to be a prominent role in the effect of UCP2 on insulin secretion, although the consequences were not always consistent. In a study published in 2009, all of the three highly congenic strain backgrounds (C57BL/6J, A/J, 129/SvImJ) Ucp2−/− mice exhibited increased oxidative stress, and the GSIS in Ucp2−/− islets of each congenic strain was significantly decreased [27]. UCP2 knockdown in INS-1E insulinoma cells improved the GSIS, and this can be annulled completely by the cell-permeative antioxidant MnTMPyP [28]. In the islets of *β*-cell-specific UCP2 knockout mice, the intracellular ROS levels were found elevated and GSIS enhanced [29]. All of these three studies described a ROS-related pathway about the role of UCP2 and GSIS. That is to say, the UCP2 knockdown will cause elevation of ROS and/or oxidative stress and then the enhancement of GSIS.

### 3.2. *UCP2* Gene Polymorphisms Are Associated with DM

A study about the associations between polymorphisms in UCP2 and UCP3 with DM2 was carried out in Korea and found that the UCP2 −5331G>A and UCP3 −2078C>T polymorphisms are susceptibility markers for DM2 among Koreans [30]. Among the other three studies, no significant association of the UCP2 −866G/A polymorphism with DM2 risk was observed [31–33]. The study about Asian Indians indicated that Ala55Val polymorphism at UCP2 and −55C/T polymorphism at UCP3 are associated with a significantly reduced risk of developing DM2 [32]. While these correlations are different between Europeans and Asian descent: neither the UCP2 Ala55Val nor the UCP3 −55C/T polymorphism showed any significant association with DM2 risk in Europeans (OR 1.04, 95% CI 0.98, 1.09 for Ala55Val; OR 1.04, 95% CI 1.00, 1.09 for −55C/T); and in contrast, a statistically significant association was observed for both polymorphisms in participants of Asian descent (OR 1.23, 95% CI 1.12, 1.36 for Ala55Val; OR 1.15, 95% CI 1.03, 1.28 for −55C/T) [33].

### 3.3. *UCP2* Gene Polymorphisms Are Associated with Other DM-Related Chronic Complications

In a study of the relationship between UCP2 polymorphisms and proliferative diabetic retinopathy (PDR), three UCP2 polymorphisms were selected (−866G/A (rs659366), Ala55Val (rs660339), and 45 bp insertion/deletion (Ins/Del)), and the haplotype [A Val Ins] was an independent risk factor for PDR in both types 1 and 2 diabetic groups [34]. Three studies focused on the relationship between −866G/A polymorphism and obesity in a Balinese population, ischemic stroke in Chinese DM2 patients, and obesity in Danes and showed a significant association between them [35–37].

### 3.4. Many Compounds or Medicines Exhibit Curative Effect on DM through Downregulated *UCP2* Gene Expression

American *Ginseng* stimulates insulin production and prevents apoptosis through downregulation of UCP2 in cultured *β*-cells [38]. Inhibited UCP2 expression by an antisense oligonucleotide can reverse diet-induced DM mice models by the effects on both insulin secretion and action [39]. MicroRNA-15a positively regulates insulin synthesis by directly targeting and inhibiting UCP2 gene expression [40]. A Chinese medicine, Kaiyuqingre formula, improves insulin secretion via decreasing the overexpression of UCP2 in cultured INS-1 cells [41]. Korean red *Ginseng* promoted the expression of insulin and downregulated the expression of UCP2 in the spontaneously diabetic Goto-Kakizaki rats [42].

Most of the studies about the role of UCP2 in DM focused on the UCP2 functions in *β*-cells, and the results indicated a deleterious effect of UCP2 on DM. As UCP2 is widely expressed in many tissues, the antioxidative activities (downregulating mitochondrial ROS generation) of this protein should be evaluated in the future.

## 4. Role of UCP3 in DM

Much fewer studies focused on the role of UCP3 in DM, for it was thought to be expressed restrictedly in skeletal muscle for a long time. UCP3 mRNA and protein levels are decreased in skeletal muscle of patients with DM2 compared to healthy control subjects. UCP3 protein content is reduced in prediabetic subjects (i.e., impaired glucose tolerance) and DM2, and eight weeks of rosiglitazone treatment significantly increases insulin sensitivity and restores skeletal muscle UCP3 protein in diabetic patients [43]. Similar to UCP2, UCP3 was found to be expressed in pancreatic *β*-cells in 2008, where it also influenced insulin secretion [44], although the physiological function of UCP3 in *β*-cells is still not known. UCP3 mRNA is expressed in human islets; the relative abundance of UCP2 mRNA was 8.1-fold higher (*P* < 0.05); immunohistochemical analysis confirmed colocalization of UCP3 protein with mitochondria in human *β*-cells. UCP2 protein expression in human islets was increased approximately 2-fold after high glucose exposure, whereas UCP3 protein expression was decreased by approximately 40% (*P* < 0.05). UCP3 overexpression improved glucose-stimulated insulin secretion. These results indicated that UCP2 and UCP3 may have distinct roles in regulating *β*-cell function; increased expression of UCP2 and decreased expression of UCP3 in humans with chronic hyperglycemia may contribute to impaired glucose-stimulated insulin secretion [44].

### 4.1. *UCP3* Gene Polymorphisms Are Associated with DM

Early in the year 2000, a study carried out French Caucasians found the association between the −55C/T polymorphism of the *UCP3* gene and DM2; the study found that subjects bearing the TT genotype have a lower risk for developing DM2 than the others do [45]. This result is the same as reported in the Asian Indians cohort [32] but differs from another study in Europeans which could not find any significant association with DM2 risk [33]. These inconsistent results may be due to the different gene background between different human species. Another report about Finnish cohort indicated that the *UCP3* gene variant rs3781907 was associated with a higher risk of DM2 [46].

### 4.2. Relationship between *UCP2-UCP3* Gene Cluster Variation and DM

The *UCP2* and *UCP3* genes are located within 8 kb of each other in a gene cluster on chromosome 11q13. Several genetic variants in the *UCP2-UCP3* gene cluster have been examined in multiple studies, including −866G/A (rs659366), Ala55Val (rs660339), a 45-bp insertion/deletion (I/D) in the 3′ untranslated region (UTR) of exon 8 in UCP2, and the −55C/T (rs1800849) polymorphism in UCP3, and the associations with obesity and/or diabetes were also reported. Many of these *UCP2* and *UCP3* gene polymorphisms (such as −866G/A, Ala55Val of UCP2, and −55C/T of UCP3) are also evaluated within *UCP2-UCP3* gene cluster variation studies; the difference between the polymorphisms and the cluster study may be just that the cluster variation studies are often focusing on many variations at the same time. A research published in 2006 evaluated the impact of the UCP2 −866G>A and UCP3 −55C>T variants on prospective risk of DM2 and found that these variations in the *UCP2-UCP3* gene cluster are associated with an increased risk of DM2 [47]. In 2008, a report focused on 14 tag single nucleotide polymorphisms (tSNP) (rs637028, rs653263, rs622064, rs2306820, rs673494, rs655717, rs643064, rs660339, rs659366, rs591758, rs668514, rs647126, rs1800006, and rs1800849), for each of the 14 tSNPs across the genomic region of the *UCP2-UCP3* gene cluster; they did not observe significant effects on DM2. However, Haplotype-based analyses suggest that a haplotype set defined by rs591758, rs668514, rs647126, and rs1800006 was significantly associated with DM2 risk in Caucasian women only, especially among those who were overweight [48]. This may be emphasizing that the DM2 development is driven by a multifactor model more than a single-factor one. In a Finnish diabetes prevention study, three variants in the *UCP2* gene, one variant in the *UCP2-UCP3* intergenic region, and five variants in the *UCP3* gene were explored [46]. The authors concluded that the genetic variations in the *UCP2-UCP3* gene cluster may act as a modifier increasing serum lipid levels and indices of abdominal obesity and may thereby also contribute to the metabolic aberrations observed in obesity and DM2. Another study analyzed other 14 tSNPs (rs622064, rs2306820, rs655717, rs660339, rs17132534, rs659366, rs668514, rs3741135, rs1626521, rs2734827, rs3781907, rs1800849, rs1685333, and rs826071) in *UCP2-UCP3* gene cluster and could not find an association of any of the 14 tSNPs tested with DM2 risk [49]. This result is similar to the other 14 tSNPs study mentioned previously, and they may both imply the same fact that the development of DM2 is a multifactor style; any one of the 14 tSNPs is not enough to affect the DM2. It is important to illustrate how the polymorphisms of *UCP* genes are involved in the development of DM, and this may help to develop new strategies for DM prevention and/or treatment.

## 5. Conclusion and Future Directions

**Figure 3:**
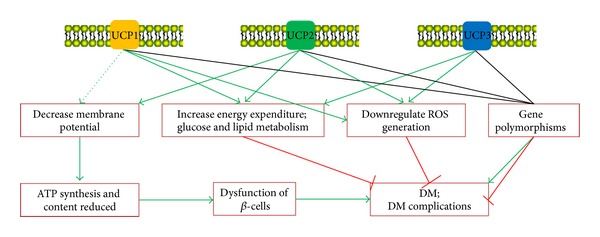
The role of UCPs on the development of DM and/or complications. UCPs may affect the development of DM through 4 aspects: decrease mitochondrial membrane potential, increase energy expenditure especially through glucose and lipid metabolisms, downregulate ROS generation, and gene polymorphisms. The green arrows represent pathways activation, the dotted green arrow means that this pathway needs to be further proved, and the red inhibition arrows represent the inhibition effect.

The present data indicated that all of the three UCPs (UCP1, UCP2, and UCP3) have some kind of relationships with the development of DM (Figure 3). As DM is a multifactorial disease, the ethnic differences, gender, genomic factors, age, nutritional characteristics, lifestyle, and even environmental factors are all related to the outcomes. The single UCP or single *UCP* gene variation may not be enough to affect the results. More importantly, the physiological function of UCPs, at least UCP2 and UCP3, is still unclear, which may limit this kind of study very much. So, it is better to keep on exploring the physiological function of UCPs in the future, and based on this, the synergetic studies on the multisite of UCPs variations may help to elucidate the relationship between UCPs and DM.

## References

[1] Maraschin JF (2012). Classification of diabetes. *Advances in Experimental Medicine and Biology*.

[2] Maiese K, Morhan SD, Zhao ZC (2007). Oxidative stress biology and cell injury during Type 1 and Type 2 diabetes mellitus. *Current Neurovascular Research*.

[3] Golbidi S, Badran M, Laher I (2012). Antioxidant and anti-inflammatory effects of exercise in diabetic patients. *Experimental Diabetes Research*.

[4] Krauss S, Zhang C, Lowell BB (2005). The mitochondrial uncoupling-protein homologues. *Nature Reviews Molecular Cell Biology*.

[5] Diano S, Horvath TL (2012). Mitochondrial uncoupling protein 2 (UCP2) in glucose and lipid metabolism. *Trends in Molecular Medicine*.

[6] Emre Y, Nübel T (2010). Uncoupling protein UCP2: when mitochondrial activity meets immunity. *The FEBS Letters*.

[7] Mehta SL, Li PA (2009). Neuroprotective role of mitochondrial uncoupling protein 2 in cerebral stroke. *Journal of Cerebral Blood Flow and Metabolism*.

[8] Nabben M, Hoeks J (2008). Mitochondrial uncoupling protein 3 and its role in cardiac- and skeletal muscle metabolism. *Physiology and Behavior*.

[9] Ramsden DB, Ho PW, Ho JW (2012). Human neuronal uncoupling proteins 4 and 5 (UCP4 and UCP5): structural properties, regulation, and physiological role in protection against oxidative stress and mitochondrial dysfunction. *Brain and Behavior*.

[10] Mailloux RJ, Harper M (2011). Uncoupling proteins and the control of mitochondrial reactive oxygen species production. *Free Radical Biology and Medicine*.

[11] Krauss S, Zhang C, Scorrano L (2003). Superoxide-mediated activation of uncoupling protein 2 causes pancreatic β cell dysfunction. *Journal of Clinical Investigation*.

[12] Geloen A, Trayhurn P (1990). Regulation of the level of uncoupling protein in brown adipose tissue by insulin. *American Journal of Physiology—Regulatory Integrative and Comparative Physiology*.

[13] Burcelin R, Kande J, Ricquier D, Girard J (1993). Changes in uncoupling protein and GLUT4 glucose transporter expressions in interscapular brown adipose tissue of diabetic rats: relative roles of hyperglycaemia and hypoinsulinaemia. *Biochemical Journal*.

[14] Jia J, Tian Y, Cao Z (2010). The polymorphisms of UCP1 genes associated with fat metabolism, obesity and diabetes. *Molecular Biology Reports*.

[15] Brondani LA, Assmann TS, Duarte GC, Gross JL, Canani LH, Crispim D (2012). The role of the uncoupling protein 1 (UCP1) on the development of obesity and type 2 diabetes mellitus. *Arquivos Brasileiros de Endocrinologia & Metabologia*.

[16] Brondani LA, de Souza BM, Duarte GC (2012). The UCP1 -3826A/G polymorphism is associated with diabetic retinopathy and increased UCP1 and MnSOD2 gene expression in human retina. *Investigative Ophthalmology & Visual Science*.

[17] de Souza BM, Brondani LA, Boucas AP (2013). Associations between UCP1 -3826A/G, UCP2 -866G/A, Ala55Val and Ins/Del, and UCP3 -55C/T polymorphisms and susceptibility to type 2 diabetes mellitus: case-control study and meta-analysis. *PLoS One*.

[18] Carroll AM, Haines LR, Pearson TW (2005). Identification of a functioning mitochondrial uncoupling protein 1 in thymus. *Journal of Biological Chemistry*.

[19] Sale MM, Hsu F, Palmer ND (2007). The uncoupling protein 1 gene, UCP1, is expressed in mammalian islet cells and associated with acute insulin response to glucose in African American families from the IRAS family study. *BMC Endocrine Disorders*.

[20] Cypess AM, White AP, Vernochet C (2013). Anatomical localization, gene expression profiling and functional characterization of adult human neck brown fat. *Nature Medicine*.

[21] Orava J, Nuutila P, Lidell ME (2011). Different metabolic responses of human brown adipose tissue to activation by cold and insulin. *Cell Metabolism*.

[22] Ma ZA, Zhao Z, Turk J (2012). Mitochondrial dysfunction and β-cell failure in type 2 diabetes mellitus. *Experimental Diabetes Research*.

[23] Jia J-J, Zhang X, Ge C-R, Jois M (2009). The polymorphisms of UCP2 and UCP3 genes associated with fat metabolism, obesity and diabetes: etiology and pathophysiology. *Obesity Reviews*.

[24] de Souza BM, Assmann TS, Kliemann LM, Gross JL, Canani LH, Crispim D (2011). The role of uncoupling protein 2 (UCP2) on the development of type 2 diabetes mellitus and its chronic complications. *Arquivos Brasileiros de Endocrinologia e Metabologia*.

[25] Dalgaard LT (2011). Genetic variance in uncoupling protein 2 in relation to obesity, type 2 diabetes, and related metabolic traits: focus on the functional -866G>A promoter variant (rs659366). *Journal of Obesity*.

[26] Zhang C, Baffy G, Perret P (2001). Uncoupling protein-2 negatively regulates insulin secretion and is a major link between obesity, β cell dysfunction, and type 2 diabetes. *Cell*.

[27] Pi J, Bai Y, Daniel KW (2009). Persistent oxidative stress due to absence of uncoupling protein 2 associated with impaired pancreatic β-cell function. *Endocrinology*.

[28] Affourtit C, Jastroch M, Brand MD (2011). Uncoupling protein-2 attenuates glucose-stimulated insulin secretion in INS-1E insulinoma cells by lowering mitochondrial reactive oxygen species. *Free Radical Biology and Medicine*.

[29] Robson-Doucette CA, Sultan S, Allister EM (2011). Beta-cell uncoupling protein 2 regulates reactive oxygen species production, which influences both insulin and glucagon secretion. *Diabetes*.

[30] Lee H, Ryu H, Shin H (2008). Associations between polymorphisms in the mitochondrial uncoupling proteins (UCPs) with T2DM. *Clinica Chimica Acta*.

[31] Heidari J, Akrami SM, Heshmat R, Amiri P, Fakhrzadeh H, Pajouhi M (2010). Association study of the -866G/A UCP2 gene promoter polymorphism with type 2 diabetes and obesity in a tehran population: a case control study. *Archives of Iranian Medicine*.

[32] Vimaleswaran KS, Radha V, Ghosh S, Majumder PP, Sathyanarayana Rao MR, Mohan V (2011). Uncoupling protein 2 and 3 gene polymorphisms and their association with type 2 diabetes in Asian Indians. *Diabetes Technology and Therapeutics*.

[33] Xu K, Zhang M, Cui D (2011). UCP2 -866G/A and Ala55Val, and UCP3 -55C/T polymorphisms in association with type 2 diabetes susceptibility: a meta-analysis study. *Diabetologia*.

[34] Crispim D, Fagundes NJR, Dos Santos KG (2010). Polymorphisms of the UCP2 gene are associated with proliferative diabetic retinopathy in patients with diabetes mellitus. *Clinical Endocrinology*.

[35] Oktavianthi S, Trimarsanto H, Febinia CA (2012). Uncoupling protein 2 gene polymorphisms are associated with obesity. *Cardiovascular Diabetology*.

[36] Chai Y, Gu B, Qiu JR (2012). The uncoupling protein 2 -866G >a polymorphism is associated with the risk of ischemic stroke in Chinese type 2 diabetic patients. *CNS Neuroscience & Therapeutics*.

[37] Andersen G, Dalgaard LT, Justesen JM (2013). The frequent UCP2 -866G>A polymorphism protects against insulin resistance and is associated with obesity: a study of obesity and related metabolic traits among 17 636 Danes. *International Journal of Obesity*.

[38] Luo JZ, Luo L (2006). American ginseng stimulates insulin production and prevents apoptosis through regulation of uncoupling protein-2 in cultured β cells. *Evidence-Based Complementary and Alternative Medicine*.

[39] de Souza CT, Araújo EP, Stoppiglia LF (2007). Inhibition of UCP2 expression reverses diet-induced diabetes mellitus by effects on both insulin secretion and action. *The FASEB Journal*.

[40] Sun L, Jiang B, Li W, Zou J, Shi Y, Liu Z (2011). MicroRNA-15a positively regulates insulin synthesis by inhibiting uncoupling protein-2 expression. *Diabetes Research and Clinical Practice*.

[41] Tong X, Song J, Zhao L, Ji H (2011). Kaiyuqingre formula improves insulin secretion via regulating uncoupling protein-2 and kATP channel. *Chinese Medical Journal*.

[42] Kim HY, Kim K (2012). Regulation of signaling molecules associated with insulin action, insulin secretion and pancreatic beta-cell mass in the hypoglycemic effects of Korean red ginseng in Goto-Kakizaki rats. *Journal of Ethnopharmacology*.

[43] Schrauwen P, Mensink M, Schaart G (2006). Reduced skeletal muscle uncoupling protein-3 content in prediabetic subjects and type 2 diabetic patients: restoration by rosiglitazone treatment. *Journal of Clinical Endocrinology and Metabolism*.

[44] Li Y, Maedler K, Haataja L (2008). UCP-2 and UCP-3 proteins are differentially regulated in pancreatic beta-cells. *PLoS ONE*.

[45] Meirhaeghe A, Amouyel P, Helbecque N (2000). An uncoupling protein 3 gene polymorphism associated with a lower risk of developing Type II diabetes and with atherogenic lipid profile in a French cohort. *Diabetologia*.

[46] Salopuro T, Pulkkinen L, Lindström J (2009). Variation in the UCP2 and UCP3 genes associates with abdominal obesity and serum lipids: the Finnish diabetes prevention study. *BMC Medical Genetics*.

[47] Gable DR, Stephens JW, Cooper JA, Miller GJ, Humphries SE (2006). Variation in the UCP2-UCP3 gene cluster predicts the development of type 2 diabetes in healthy middle-aged men. *Diabetes*.

[48] Hsu Y, Niu T, Song Y, Tinker L, Kuller LH, Liu S (2008). Genetic variants in the UCP2-UCP3 gene cluster and risk of diabetes in the women’s health initiative observational study. *Diabetes*.

[49] Zee RYL, Ridker PM, Chasman DI (2011). Mitochondrial uncoupling protein gene cluster variation (UCP2-UCP3) and the risk of incident type 2 diabetes mellitus: the Women’s Genome Health Study. *Atherosclerosis*.

